# FBXL16 Promotes Endometrial Progesterone Resistance via PP2A^B55*α*^/Cyclin D1 Axis in Ishikawa

**DOI:** 10.1155/2022/7372202

**Published:** 2022-09-05

**Authors:** Haoen Liu, Li Han, Liyan Zhong, Xiaodan Zhuang, Yan Peng

**Affiliations:** ^1^Wujin Hospital Affiliated with Jiangsu University, Changzhou 213017, China; ^2^The Wujin Clinical College of Xuzhou Medical University, Changzhou 213017, China; ^3^Nilek County People's Hospital, XinJiang YiLi Kazak Autonomous Prefecture, Ili Kazakh Autonomous Prefecture 835000, China; ^4^Nantong University, Nantong 226019, China

## Abstract

**Background:**

F-box proteins are essential components of the E3 ubiquitin ligases which are involved in the regulation of almost all life activities such as cell cycle, proliferation, and apoptosis, which have become an important research and drug target. However, there are few studies on F-box and leucine-rich repeat protein 16 (FBXL16) in endometrial carcinoma.

**Methods:**

Clinical samples were collected for determining the correlation between FBXL16 and endometrial carcinoma. Cells were screened and established with Ishikawa cells which proved the fundamental role of FBXL16 in regulating cell proliferation and cell cycle. The MPA-resistant endometrial carcinoma cell line Ishikawa/MPA was established. FBXL16, PP2A^B55*α*^, and cyclin D1 were analyzed separately in MPA sensitive and resistant Ishikawa cells in vitro and in vivo.

**Results:**

The high expression of FBXL16 was positively correlated with MPA resistance and poor prognosis of endometrial cancer. MPA tolerance of endometrial cancer cells was inhibited by knockdown of FBXL16 in DNA content assessment, CCK-8, and colony formation. It was confirmed that FBXL16 inhibited the activity of substrate PP2A^B55*α*^ by binding to PP2A, reduced the phosphorylation level at Thr308 site of AKT1, inhibited the expression of GSK-3*β*, and thus led to a significant decrease in the phosphorylation level of cyclin D1, which prevented the ubiquitination recognition and degradation of cyclin D1.

**Conclusion:**

In our experiments, FBXL16 binds PP2A to promote the dephosphorylation of Thr286 site of cyclin D1 via AKT1/GSK3*β*/cyclin D1 pathway, which is required for resisting the ubiquitination degradation and enhances the MPA resistance of Ishikawa.

## 1. Introduction

In recent years, endocrine therapy is mainly used to preserve the reproductive function of women patients with endometrial cancer. Among them, the success rate is 57%-75% [1, 2]. It is worth noting that about 40% of patients are not sensitive or resistant to progesterone, which limits the clinical medication of this population [3]. Therefore, exploring the mechanism of progesterone resistance in endometrial cancer has great guiding significance for clinical diagnosis and treatment.

So far, many studies have focused on endometrial cancer, trying to understand the functions of genes and proteins in tumor proliferation [4–6]. Among these studies, the ubiquitin-proteasome system, a highly selective process for degrading proteins with the 26s proteasome complex, has been a hot topic [7–9]. FBXL16 (F-Box and Leucine-Rich Repeat Protein 16) is a poorly studied F-box protein that brings the substrate to the E3 ligase complex and mediates the subsequent degradation [10–12]. PP2A (Protein Phosphatase 2), usually defective or deregulated in many types of cancer, is a major heterotrimeric serine/threonine phosphatase that consists of one structural (PP2A/A), one catalytic (PP2A/C), and one regulatory (PP2A/B) subunit [13–15]. Honarpour et al. found that FBXL16 regulated the origin of FLK+ progenitor cells by binding to PP2A^B55a^ protein and played an important role in the differentiation of embryonic stem cells [11]. Although substantial evidence pointed out that PP2A probably acted as a tumor suppressor, however, the function of PP2A in endometrial carcinoma and the process of progesterone resistance are still unclear.

Therefore, based on the prediction of the gepia2 database and the mRNA level of clinical patients' tumor tissues, we explored the role of FBXL16 in affecting cell proliferation and promoting the MPA (mycophenolic acid) resistance of endometrial cancer cells in vitro and in vivo. FBXL16 was upregulated in endometrial carcinoma tissues and many kinds of endometrial cell lines. Compared to the parental cells, the expression of FBXL16 increased remarkably in the MPA-resistant Ishikawa cells. Furthermore, FBXL16 promoted the MPA resistance of Ishikawa cells by interacting with PP2A^B55*α*^ and enhancing the stability of cyclin D1 in endometrial carcinoma. These findings indicate that FXBL16 may be a potential diagnostic marker and therapeutic target in the MPA resistance process of endometrial carcinoma.

## 2. Materials and Methods

### 2.1. Cell Culture and MPA-Induced Treatment

Endometrial cancer cell lines, HEC-1A, HEC-1B, AN3-CA, RL95-2, Ishikawa, and KLE, and immortalized endometrial epithelium cell line, EEC-1, were purchased from Bluefcell (Shanghai) Biotechnology Development Co., Ltd. Cells were cultured in Dulbecco's Modified Eagle Medium (Gibco, 11965-092) with a supplement of 10% fetal bovine serum (Gibco, 26140-095) and 1% antibiotics (Gibco, 15070-063). Cells were maintained at 37°C with 5% CO_2_ in a humidified environment.

### 2.2. Animal Models

Balb/c nude mice (female, age 4-6 weeks) were purchased from Jingling Hospital (Nanjing, China) and maintained in a pathogen-free facility. MPA-resistant cells with shFBXL16 transfected (Ishikawa-MPA/shFBXL16) and shNC (Ishikawa-MPA/shNC) were subcutaneously injected into the right armpit region of balb/c nude mice separately (groups = 2, *n* = 6). The animal studies were approved by the Institutional Animal Care and Use Committee of Wujin Hospital Affiliated with Jiangsu University. Animal studies were performed in accordance with institutional guidelines.

### 2.3. FBXL16 shRNA Transfection

Cell transfection with FBXL16 shRNA was conducted according to the previous work. Briefly, Lipofectamine 3000 (Invitrogen, USA) was used for performing transfections according to the manufacturer's protocols. The MPA-resistant Ishikawa cells were treated with the pTRIPZ inducible lentiviral shRNA targeting FBXL16, using an empty pTRIPZ lentiviral vector as the negative control. All transfection experiments were carried out according to the manufacturer's instructions (Horizon Discovery).

### 2.4. Quantitative Real-Time Polymerase Chain Reaction (RT-qPCR)

Total RNA was extracted from cells and tissues with TRIzol reagent (Invitrogen, USA). Prime Script RT master mix (Takara, Japan) was added for reverse transcription of total RNA. SYBR Premix Ex Tag™ II (Takara, Japan) was used for the examination of gene expression based on the 2^-*ΔΔ*CT^ method. GAPDH was employed as the internal reference. Primers of FBXL16, cyclin D1, cyclin E1, and GAPDH were listed in the Supplementary Table S1. Three independent assays were requested.

### 2.5. Western Blot Analysis

Ishikawa cells were treated with EBC buffer (Roche Diagnostics) and 1 mM phosphatase inhibitor mixture II (Sigma-Aldrich). SDS-PAGE was applied by transferring the proteins onto nitrocellulose membranes. 5% nonfat milk in PBS with Tween 20 was subsequently utilized to block the membranes. Afterward, the membranes were incubated with primary antibodies against GAPDH (CST, Cat. No. 5174S, 1 : 1000), FBXL 16 (Merck, Cat. No. SAB2103554, 1 : 1000), PP2A-^A*α*/*β*^ (Santacruz, Cat. No. sc-13600, 1 : 800), PP2A^B55a^ (Santacruz, Cat. No. sc-81606, 1 : 800), PP2A^C^ (Santacruz, Cat. No. sc-13601, 1 : 800), cyclin D1 (Abcam, Cat. No. ab182858, 1 : 3000), cyclin D1 phosphor T286 (Abcam, Cat. No. ab62151, 1 : 1000), AKT1 phosphor T308 (Abcam, Cat. No. ab278565, 1 : 1000), GSK3*β* (Abcam, Cat. No. ab32391, 1 : 2000), and ki-67 (Abcam, Cat. No. ab184787, 1 : 1000) overnight at 4°C. After being washed with 1× TBST, the membranes were incubated with respective secondary antibodies conjugated with horseradish peroxidase for 1 h at room temperature. The protein bands were visualized with Immobilon™ Western Chemiluminescent HRP Substrate (Millipore Corporation, Cat. No. WBKLS0500), and the images were captured on the visualization instrument Tanon-5200 (Tanon, China).

### 2.6. Colony Forming Assays

To assess effects on colony formation, transfected Ishikawa cells and negative control were seeded at 500 cells/mL in 6-well plates for growing for about 14 days. Finally, cells were stained with crystal violet. Colonies containing ≥50 cells were counted.

### 2.7. Annexin V Detection for Apoptosis

Cells were collected and placed into 6-well plates for siRNA treatment. The cells were then harvested at a concentration of1 × 10^6^cells/mL. A flow cytometer was used following the instruction (BD Biosciences, Franklin Lakes, N, USA), and the Annexin V-APC/7-AAD double staining kit was purchased from Jiangsu KeyGEN BioTECH. After 15 min of staining, samples were analyzed by flow cytometry. Each group of samples was repeated independently three times.

### 2.8. Cell Cycle Assessment

DNA content detection of the treated cells was carried out in the BD Calibur procedure, and the DNA content assay was purchased from Jiangsu KeyGEN BioTECH. After two hours in cold 70% (*v*/*V*) ethanol solution, cell samples were stained by following the instruction of the assay. Each group of samples was repeated independently three times.

### 2.9. Cell Viability Assay

For cell viability analysis, cells were plated in a 96-well plate at 5000 cells per well, approximately. The following day, cells were exposed to different concentrations of MPA, and after 72-hour exposure, cell survival was assessed with the Cell Counting Kit-8 in accordance with the recommended guideline (KeyGEN BioTECH, Nanjing, China). The half-maximal inhibitory concentration (IC_50_) curves of MPA resistance in Ishikawa cells were calculated by the GraphPad 7.0 software.

### 2.10. 5-Ethynyl-2′-deoxyuridine (EdU)

The effects of FBXL16 on cell proliferation were analyzed by EdU staining (Riobio, Guangzhou, China). Briefly, 2000 cells were placed into 96-well plates and incubated with 10 nM EdU, for 24 hours. Afterward, PBS was used to wash the wells, and the cells were fixed with 4% paraformaldehyde and stained according to the manufacturer's instructions. Later, the images were recorded with a fluorescence microscope (Leica DMI6000B).

### 2.11. Ubiquitination Assay

To determine the ubiquitination level in vivo, Ishikawa cells were dissolved and boiled in ubiquitination buffer containing 1% SDS at 95°C for 10 min. The denatured cell lysates were then mixed with an SDS-negative control buffer until the final concentration of SDS was 0.2% (*w*/*V*). The lysates were uploaded in an SDS-PAGE and immunoblotted with anti-E3 ubiquitin ligase (1 : 3000) and anticyclin D1 (1 : 1000), respectively.

### 2.12. Immunoprecipitation

For analysis of FBXL16 and PP2A interaction, Ishikawa cells were treated with 100 nM nocodazole for 4 hours and lysed in NP-40 lysis buffer. Antibodies to PP2A/C subunits (Santa Cruz, sc-80665), PP2A^B55-*α*^ (Santa Cruz, sc-81606), PP2A/A subunits (Santa Cruz, sc-56954), and FBXL16 (Thermo Fisher, PA5-58818) were incubated with cell extracts at 4°C for 16 hours. Antibody-protein complex was bonded to protein A/G beads and then subjected to western blot after washing 3 times with NP40 lysis buffer.

### 2.13. Protein Stability

To measure protein stability, Ishikawa/MPA with shRNA-NC and shFBXL16 cells was treated with cycloheximide (CHX, working concentration at 100 *μ*g/mL) during indicated times. The expression of cyclin D1 and cyclin E was measured by western blot analysis.

### 2.14. HE and IHC Staining

The tissues were collected and then soaked in 4% formalin. Later, they were dehydrated and embedded in paraffin. A microtome was used to acquire sections with a thickness of 5 *μ*m. Subsequently, H-E staining was conducted. Sections for IHC were stained with FBXL16 and Ki67.

### 2.15. Statistical Analysis

Data and graphics were reported using GraphPad Prism (v9.0). All data were performed in three independent repetitions, and the data from each group are presented as mean ± SD. Group comparisons were assessed by one-way ANOVA or Fisher's exact test. *P* < 0.05 was used to determine the statistical significance.

## 3. Results

### 3.1. FBXL16 Is Highly Expressed in MPA-Resistant Endometrial Cancer Tumors

**Figure 1:**
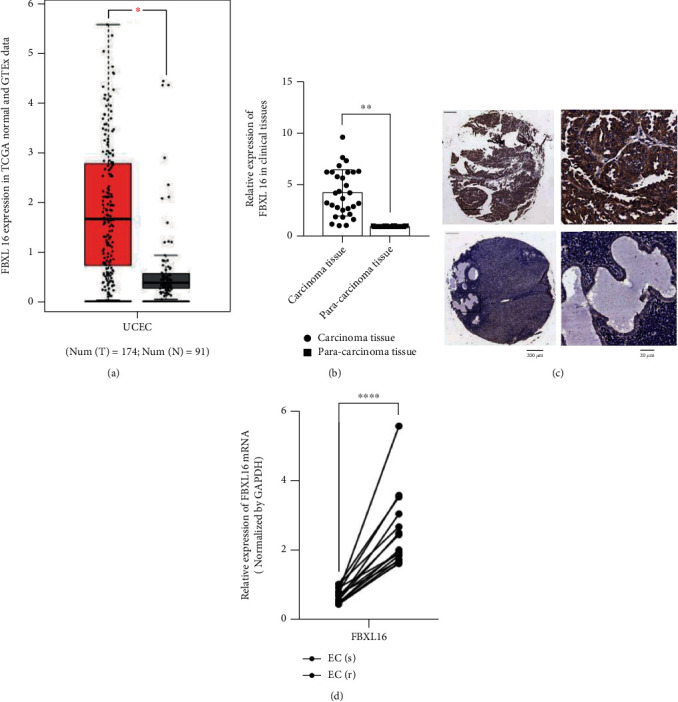
FBXL16 is highly expressed in MPA-resistant endometrial cancer tumors. (a) Box plot from GEPIA2 database indicated the expression of FBXL16 in UCEC tissues. (b) RT-qPCR examined the expression of FBXL16 in endometrial cancer tissues and paracancer tissues (group = 2, *n* = 30). (c) Immunohistochemistry analysis of FBXL16 expression in endometrial cancer tissues and paracancer tissues. (d) RT-qPCR assessed the expression of FBXL16 in EC/r and EC/s (group = 2, *n* = 15). Results are presented as mean ± SD. ^∗^*P* < 0.05. ^∗∗^*P* < 0.01. ^∗∗∗^*P* < 0.001.

Based on the TCGA database, FBXL16 presented a high expression level in 174 cases of Uterine Corpus Endometrial Carcinoma (UCEC) tissues relative to 91 cases of normal tissues (Figure 1(a)). Therefore, the expression profile of FBXL16 in cancer and adjacent tissues of endometrial cancer associated with estrogen resistance was further investigated by RT-PCR. Clinical samples were collected from 30 clinical patients for FBXL16 profile analysis. As illustrated in Figure 1(b), an increased mRNA level of FBXL16 could be observed in the endometrial cancer group while the normal control showed a very low expression. It implied that the expression of FBXL16 in normal tissues and endometrial cancer tissues was significantly different. The carcinoma tissues and pare-carcinoma tissues were furtherly conducted immunohistochemistry (IHC) to show the expression of FBXL16. And a statistical analysis on the collected samples was performed. As shown in Figure 1(c), the expression level of FBXL16 protein in the endometrial cancer patient group was significantly higher than that of the normal healthy group (∗∗*P* < 0.01). And in Figure 1(d), the mRNA expression level of FBXL16 was remarkably higher in MPA-sensitive endometrial cancer tissues (EC/s) than that in MPA-resistant endometrial cancer tissues (EC/r). These results suggested that FBXL16 was commonly and highly expressed in patients with endometrial cancer, indicating a role of FBXL16 in the pathogenesis of endometrial cancer.

### 3.2. FBXL16 Knockdown Inhibited Cell Proliferation and MPA Resistance of Ishikawa

**Figure 2:**
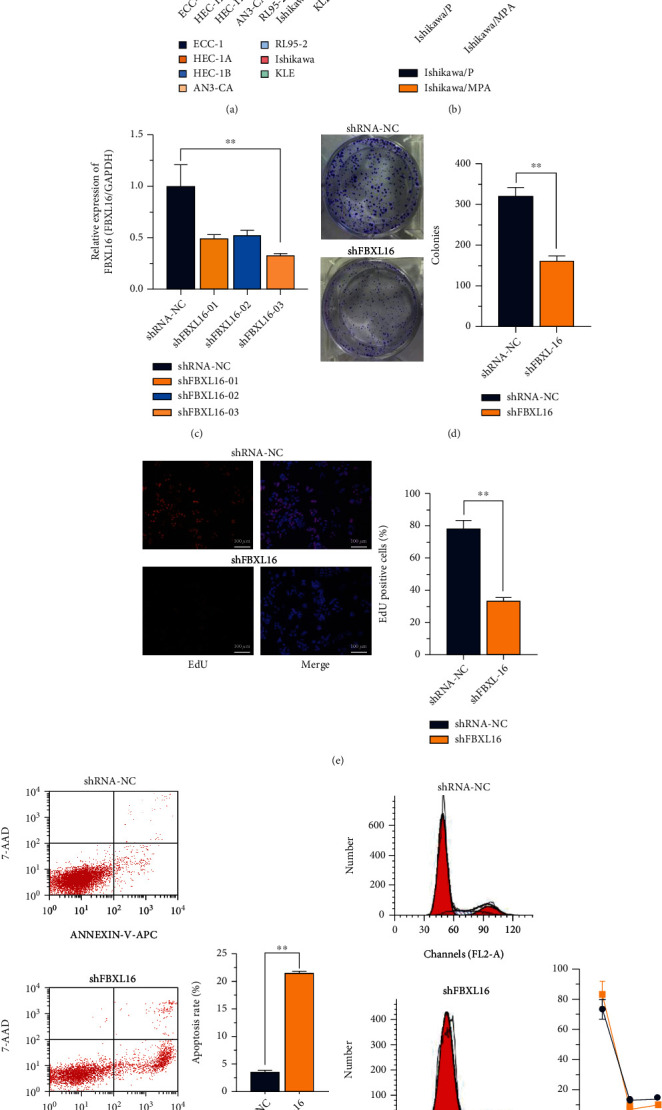
FBXL16 knockdown inhibited cell proliferation and MPA resistance of Ishikawa. (a) RT-qPCR examined the expression of FBXL16 in endometrial cancer cell lines in comparison with human immortalized endometrial epithelial cells (EEC-1). (b) RNA level of FBXL16 validated by RT-qPCR in parent Ishikawa cells (Ishikawa/P) and Ishikawa cells resistant to MPA (Ishikawa/MPA). (c) Transfection efficiency of shFBXL16 in transfected Ishikawa/MPA cells by RT-qPCR. (d) Colony formation assay of Ishikawa/MPA with the shRNA-NC group or shFBXL16 group. (e) EdU incorporation assay of Ishikawa/MPA with the shRNA-NC group or shFBXL16 group. (f) Apoptosis detection of Ishikawa/MPA with the shRNA-NC group or shFBXL16 group by Annexin V-APC/7-AAD method. (g) DNA content detection of Ishikawa/MPA with the shRNA-NC group or shFBXL16 group by PI staining. Results are presented as mean ± SD. ^∗^*P* < 0.05. ^∗∗^*P* < 0.01. ^∗∗∗^*P* < 0.001.

As the qPCR result showed in Figure 2(a), Ishikawa was identified with the highest expression of FBXL16 to be the appropriate cell line in this study for further verifying the internal mechanism of the association in vitro, and MPA-resistant cell line was established by continuous administration of increasing concentration (0.1-128 *μ*g/mL) MPA inducement to Ishikawa cells. Compared with the parent Ishikawa (Ishikawa/P), the MPA resistance ratio was about 3.3 times higher in MPA-resistant Ishikawa (Ishikawa/MPA) (see details in Supplementary Figure S1). FBXL16 was highly increased in the Ishikawa/MPA group compared to the Ishikawa/P group by qPCR validation (Figure 2(b)). To clarify the biological function of FBXL16, three different shRNA against FBXL16 (designated as shRNA 01-03) were utilized to knockdown FBXL16 in Ishikawa/MPA. shRNA-3 exhibited a significant decrease in endogenous FBXL16 expression (Figure 2(c)); therefore, we selected shFBXL16-03 and shRNA-NC for lentivirus packaging to screen the stable transfected Ishikawa/MPA cell line (Ishikawa-MPA/shFBXL16) and its negative control cell (Ishikawa-MPA/shNC) for the subsequent experiments. Next, cell function assays were subsequently implemented to determine the function of FBXL16 in Ishikawa cells. As indicated in colony formation assays, the number of colonies was reduced after FBXL16 knockdown in Ishikawa-MPA/shFBXL16 compared to Ishikawa-MPA/shNC (Figure 2(d)). Consistently, the EdU positive rate of stained cells was lessened as FBXL16 was downregulated in Ishikawa-MPA/shFBXL16 (Figure 2(e)). In addition, FBXL16 knockdown led to an augment on apoptosis rate in the Ishikawa-MPA/shFBXL16 group, by Annexin V-APC/7-AAD staining (Figure 2(f)) and arrestment in G1 phase by DNA content detection (Figure 2(g)) by flow cytometry analyses. The cell growth curve and IC_50_ of MPA in Ishikawa-MPA/shFBXL16 and Ishikawa-MPA/shNC were listed in Supplementary Figure S2-1 and S2-2. All these data revealed a positive association of FBXL16 in cell cycle regulation and tumor proliferation promotion in MPA-resistant endometrial cancer cell, Ishikawa/MPA.

### 3.3. FBXL16 Stabilized Cyclin D1 Protein by Indirect Interactions

**Figure 3:**
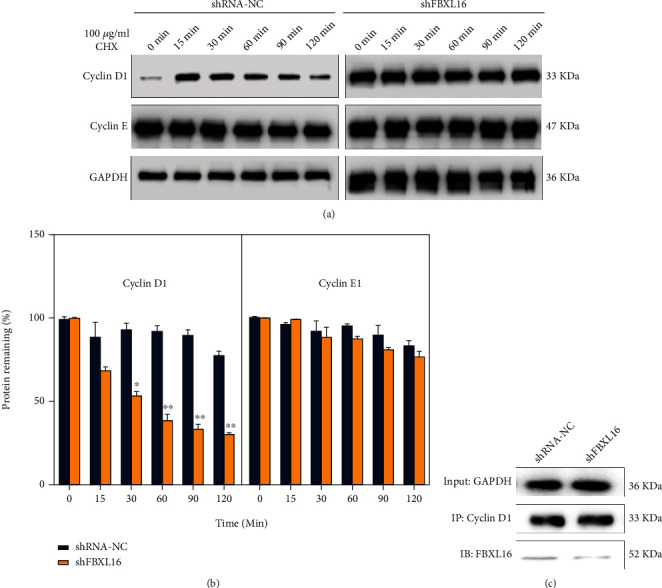
FBXL16 stabilized cyclin D1 protein by indirect interactions. (a, b) Ishikawa/MPA cells with shRNA-NC or shFBXL16 were pretreated with CHX for 90 min, and protein expression of cyclin D1 and cyclin E was analyzed by western blot at indicated times (left) and quantitatively analyzed (right). (c) Coimmunoprecipitation of FBXL16 and cyclin D1 in Ishikawa/MPA cells with the shRNA-NC or shFBXL16 group. Results are presented as mean ± SD. ^∗^*P* < 0.05. ^∗∗^*P* < 0.01. ^∗∗∗^*P* < 0.001.

In view of the above results, we further verified whether cyclin D and cyclin E were also decreased after FBXL16 knockdown in Ishikawa/MPA [16–19]. As expected, cyclin D1 and cyclin E1 were also significantly decreased with FBXL16 knockdown in real-time PCR (∗∗*P* < 0.05) (Supplementary Figure S3). We doubted whether FBXL16, to some extent, was responsible for the stability of cyclin D1 and cyclin E. Therefore, we conducted the protein stability test of cyclin D1 and cyclin E by 100 *μ*g/mL CHX treatment by a processing time for a series of gradient (0 min, 30 min, 60 min, 90 min, and 120 min), respectively. It was found that compared to the shFBXL16 negative control group (Ishikawa-MPA/shNC), cyclin D1 of Ishikawa-MPA/shFBXL16 appeared much less stable over time, with a significant difference of groups (∗∗*P* < 0.05); meanwhile, the stability of cyclin E also has dropped in the Ishikawa-MPA/shFBXL16 group, but with no significant difference (Figures 3(a) and 3(b)). However, the immunoprecipitation results of FBXL16 and cyclin D1 were negative both in the shFBXL16 and shRNA-NC groups of Ishikawa, suggesting that FBXL16 may not directly bind cyclin D1 to affect protein stability (Figure 3(c)). Based on the above results, we concluded that FBXL16 may regulate the cell cycle of Ishikawa and promote tumor proliferation through some indirect regulations to stabilize cyclin D1 at the protein level.

### 3.4. FBXL16 Dephosphorylated and Stabilized Cyclin D1 Protein by Regulating Akt1/GSK-3*β* Signaling Pathway in a PP2A^B55*α*^ Manner

Based on our experimental results and previous studies reported, FBXL16 can regulate the phosphorylation activity of PP2A substrate in combination with PP2A/B/C, and FBXL16 may also have some antagonistic effect on FBW7 to inhibit the ubiquitination of SCF^FBW7^ substrate [10, 20]. Therefore, we boldly envisage whether FBXL16 may stabilize or promote the expression of cyclin D1 by inhibiting the ubiquitination or phosphorylation of cyclin D1. The preliminary immunoprecipitation detection results showed that there was no binding FBW7 positive band (Supplementary Figure S4) in either the negative control or the Ishikawa/MPA after FBXL16 knockdown. Therefore, we carried out further studies on the correlation between FBXL16, PP2A/B/C, and cyclin D1 in ubiquitination and phosphorylation.

**Figure 4:**
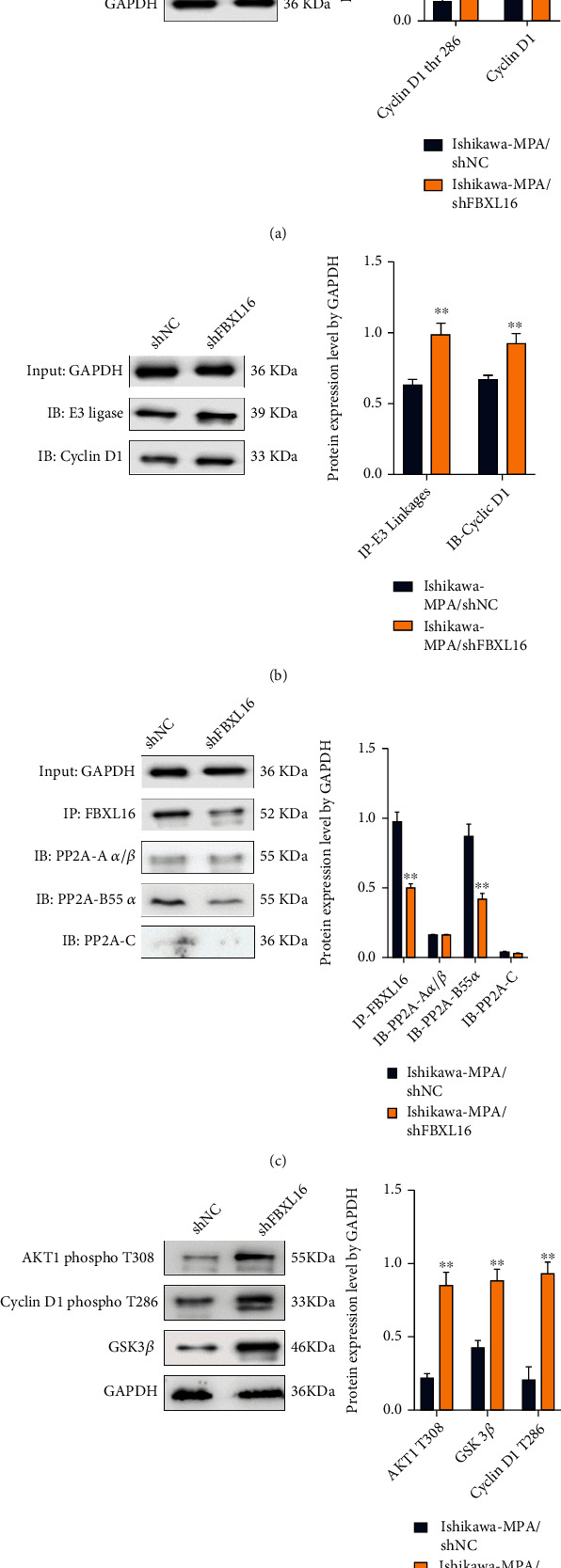
FBXL16 dephosphorylated cyclin D1 by regulating Akt1/GSK-3*β* signaling pathway via binding PP2A^B55*α*^. (a) Western blot analyses of cyclin D1 and phosphor-cyclin D1 (T286) in Ishikawa/MPA with shNC and shFBXL16 cells. (b) Coimmunoprecipitation of E3 ligase and cyclin D1 in Ishikawa/MPA cells with the shRNA-NC or shFBXL16 group. (c) Coimmunoprecipitation of FBXL16, PP2A-A *α*/*β*, PP2A-B55*α*, and PP2A-C in Ishikawa/MPA cells with the shRNA-NC or shFBXL16 group. (d) Western blot analyses of phosphor-cyclin D1 (T286) and GSK3*β*/AKT1 pathway in Ishikawa/MPA with shNC and shFBXL16 cells. Results are presented as mean ± SD. ^∗^*P* < 0.05. ^∗∗^*P* < 0.01. ^∗∗∗^*P* < 0.001.

Compared with the shFBXL16 negative control group, the phosphorylation level of cyclin D1 showed that the thr286 site was significantly upregulated in the FBXL16 knockdown group (Figure 4(a)), and the corresponding E3 ubiquitination level was also upregulated (Figure 4(b)), which was consistent with previous reports; the Thr286 site may be related to the ubiquitination recognition site of cyclin D1 [21], and the phosphorylation of this site may lead to the E3 ligase recognition of the protein, resulting in ubiquitination degradation. Next, we further verified the changes of phosphorylation substrates of three main subunits of PP2A by IP and western blot, and PP2A^B55a^ was confirmed to bind to FBXL16 in IP products (Figure 4(c)). The B55*α* subunit of protein phosphatase 2A (PP2A) has been implicated in AKT dephosphorylation [22–25]. AKT1/GSK-3*β*/cyclin D1 pathway was subsequently examined by phosphorylated protein level in western blot. The phosphorylation level of Akt1 at thr308 site and cyclin D1 at thr286 was significantly improved in Ishikawa-MPA/shFBXL16 compared to the shNC group; meanwhile, the expression of GSK-3*β* was also greatly upregulated in the knockdown group (∗∗*P* < 0.05) (Figure 4(d)). These data indicated that FBXL16 dephosphorylated cyclin D1 by regulating Akt1/GSK-3*β* signaling pathway via binding PP2A^B55*α*^.

### 3.5. FBXL16 Promotes MPA Resistance and Proliferation of Endometrial Cancer Tumors In Vivo

**Figure 5:**
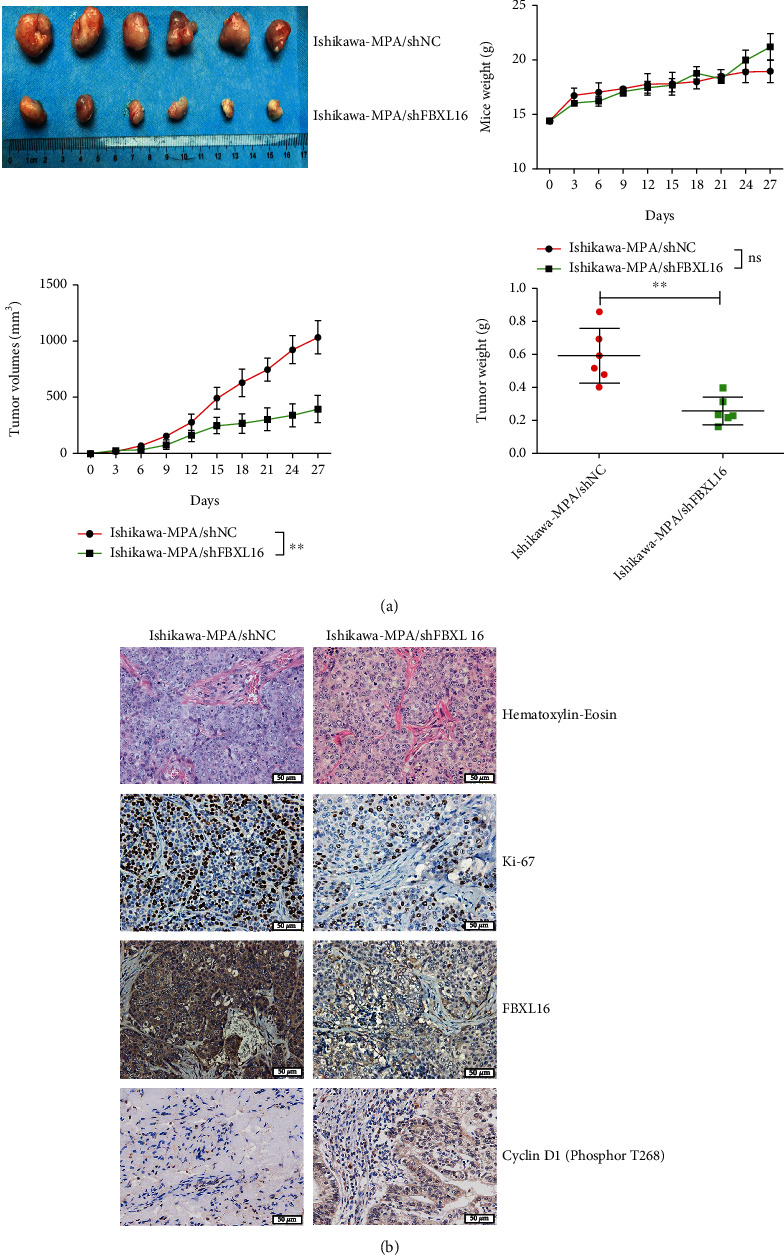
FBXL16 promotes MPA resistance and proliferation of endometrial cancer tumors in vivo. (a) The xenografts from nude mice inoculated in Ishikawa-MPA/shNC and Ishikawa-MPA/shFBXL16 cells. The tumor volumes in nude mice and mouse weight were determined for 27 days. Mean tumor weights at day 27 were determined. (b) H-E staining and IHC analysis of Ki-67, FBXL16, and phosphor-cyclin D1 (T268) were performed in the tumor tissue sections of Ishikawa-MPA/shNC and Ishikawa-MPA/shFBXL16. Data are represented as mean ± SD (*n* = 5 per group).

Finally, to further explore the role of FBXL16 in proliferation and MPA-resistance of endometrial cancer in vivo, tumor bearing model on nude balb/C mice was established. The mice were subsequently injected with Ishikawa with shNC lentiviral vector (Ishikawa-MPA/shNC) or with shFBXL16 lentiviral vector (Ishikawa-MPA/shFBXL16), compared to which were directly injected with Ishikawa/P and Ishikawa/MPA. Nude mice were sacrificed after 28 days of tumor bearing, and tumor volume and weight were analyzed to determine tumor growth rate. Tumor volume of the Ishikawa-MPA/shFBXL16 group was similar to that of Ishikawa/P, both of which were smaller than that in the Ishikawa/MPA and Ishikawa-MPA/shNC groups (Figure 5(a)). Moreover, HE analysis and Ki67, FBXL16, and cyclin D1 (phosphor T268) were analyzed by IHC staining (Figure 5(b)). In summary, our results demonstrated that FBXL16 promoted MPA resistance of Ishikawa cell by PP2A^B55*α*^ interaction and AKT1/GSK3*β*/cyclin D1 pathway in endometrial carcinoma, while knockdown of FBXL16 can reverse the MPA resistance of Ishikawa.

## 4. Discussion

Although the F-box proteins have been recognized to play an important role in cell proliferation and tumor generation [26–28], the function of FBXL16 in endometrial carcinoma is virtually unknown. Compared to other F-box proteins, FBXL16 may have distinct functions since it does not form a stable ligase complex [10–12, 29, 30]. In our experiment, FBXL16 was demonstrated to enhance MPA resistance in endometrial cancer cells. FBXL16 could promote cell proliferation and accelerate the G1-phase, which contributed to tumor generation in nude mice. Results in vivo also demonstrated that this effect could be reversed through inhibiting the expression of FBXL16. Moreover, the immunohistochemical results showed the highly positive correlation between Ki67 and FBXL16, implying the oncogenic role of FBXL16 in endometrial carcinoma. These findings also indicated the great potential of FBXL16 as a therapeutic target in endometrial carcinoma and other tumors.

FBXL16/PP2A^B55*α*^ promoted the dephosphorylation of Akt1 and downregulated the expression of GSK3*β*, which inhibited the phosphorylation and ubiquitylation level of cyclin D1 in the downstream signaling pathway. The abnormal expression of FBXL16 facilitated the MPA resistance of endometrial cancer by stabling the cyclin D1 protein; however, the proteasome-dependent degradation of cyclin D1 recognized by ubiquitinated E3 ligase was not the only one mechanism of MPA resistance. Further study on transcription profiling or protein profiling of FBXL16 overexpression or knockdown in endometrial cancer cells could be expected in the future.

It is worth mentioning the limitations of the coimmunoprecipitation experiment. Due to the sensitivity of coimmunoprecipitation and expression abundance of proteins, direct binding of FBXL16 to cyclin D1 was not found in this study, but the possibility of binding of FBXL16 to cyclin D1 could not be completely denied.

This study was to verify whether there is a direct binding combined with PP2A^B55*α*^ and cyclin D1 in mammal cells at the beginning; after all in the yeast, researchers can fully demonstrate the direct combining the homologous analogue PP2A^B55*α*^ with cyclin D1. But unfortunately, in endometrial carcinoma model in this study, the two also have no positive in combination with the results. The regulatory mechanism of cyclin D1 remains to be further explored in the future.

Our work revealed that the abnormal expression of FBXL16 in Ishikawa cells led to the inhibition of GSK3*β*-dependent cyclin D1 degradation and ultimately the promotion of MPA resistance in endometrial carcinoma. Inhibiting the expression of FBXL16 may be new direction for progesterone treatment resistance in endometrial carcinoma.

## Data Availability

The data used to support the findings of this study are included within the article.

## Supplementary Materials

Supplementary Materials Figure S1: MPA IC_50_ of Ishikawa cells determined by CCK-8 assays. Figure S2-1: the cell growth curve of Ishikawa-MPA/shFBXL16 and Ishikawa-MPA/shNC cells by CCK-8 assays. Figure S2-2: the IC_50_ of MPA in Ishikawa-MPA/shFBXL16 and Ishikawa-MPA/shNC by CCK-8 method. Figure S3: the mRNA level of FBXL16, cyclin D1, and cyclin E1 in Ishikawa-MPA/shFBXL16 and Ishikawa-MPA/shNC cells performed by real-time PCR. Figure S4: the protein expression of FBW7 in Ishikawa-MPA/shFBXL16 and Ishikawa-MPA/shNC cells by immunoprecipitation detection. Table S1: primers of genes for qRT-PCR used in this study.
