# Supplementary material for: FBXL16 Promotes Endometrial Progesterone Resistance via PP2AB55α/Cyclin D1 Axis in Ishikawa
Source: J Immunol Res. 2022 Sep 5;2022:7372202. doi: 10.1155/2022/7372202 (PMC9467819; doi:10.1155/2022/7372202)
Supplement: Supplementary Materials — Figure S1: MPA IC50 of Ishikawa cells determined by CCK-8 assays. Figure S2-1: the cell growth curve of Ishikawa-MPA/shFBXL16 and Ishikawa-MPA/shNC cells by CCK-8 assays. Figure S2-2: the IC50 of MPA in Ishikawa-MPA/shFBXL16 and Ishikawa-MPA/shNC by CCK-8 method. Figure S3: the mRNA level of FBXL16, cyclin D1, and cyclin E1 in Ishikawa-MPA/shFBXL16 and Ishikawa-MPA/shNC cells performed by real-time PCR. Figure S4: the protein expression of FBW7 in Ishikawa-MPA/shFBXL16 and Ishikawa-MPA/shNC cells by immunoprecipitation detection. Table S1: primers of genes for qRT-PCR used in this study. [file 7372202.f1.pdf]

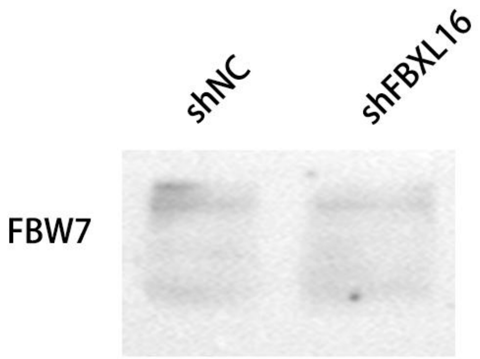

Figure S1. MPA IC_{50} of Ishikawa cells determined by CCK-8 assays.

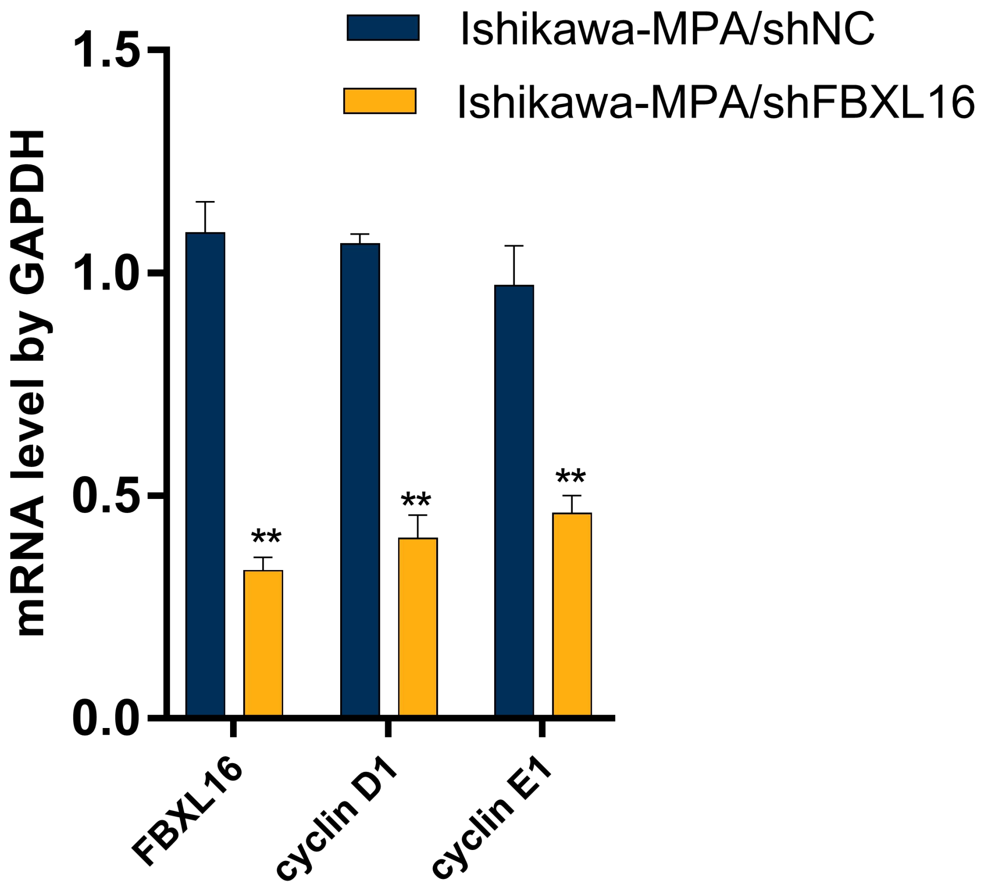

Figure S2-1. The cell growth curve of Ishikawa-MPA/shFBXL16 and Ishikawa-MPA/shNC cells by CCK-8 assays.

Figure S5. Primers of genes for qRT-PCR used in this study.

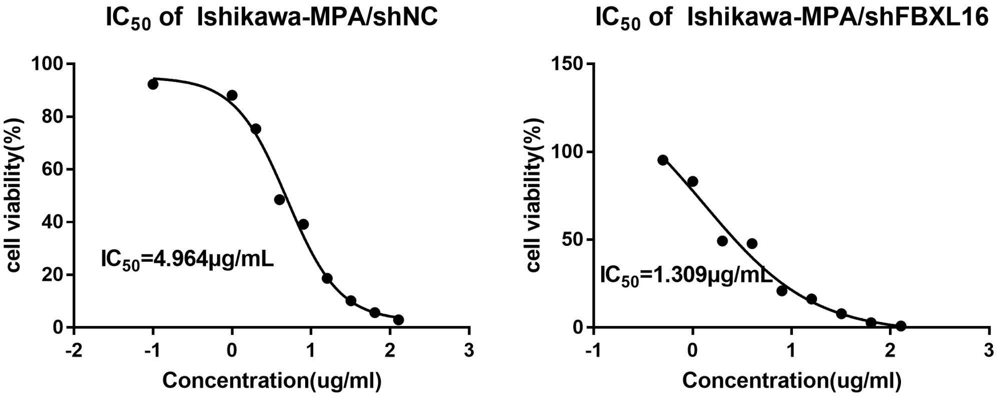

Figure S2-2. The IC_{50} of MPA in Ishikawa-MPA/shFBXL16 and Ishikawa-MPA/shNC by CCK-8 method.

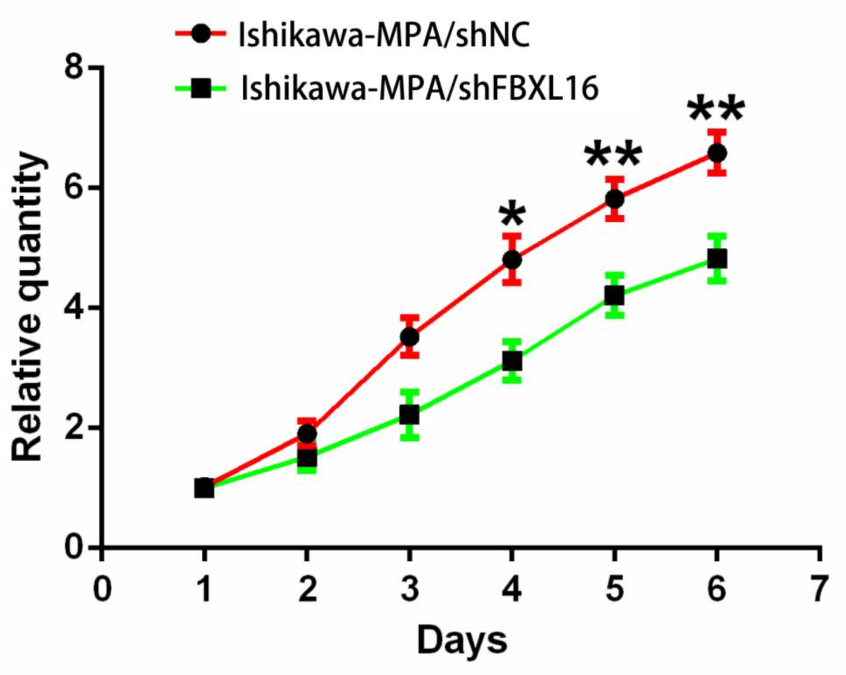

Figure S3. The mRNA level of FBXL16, cyclin D1 and cyclin E1 in Ishikawa-MPA/shFBXL16 and Ishikawa-MPA/shNC cells performed by real-time PCR.

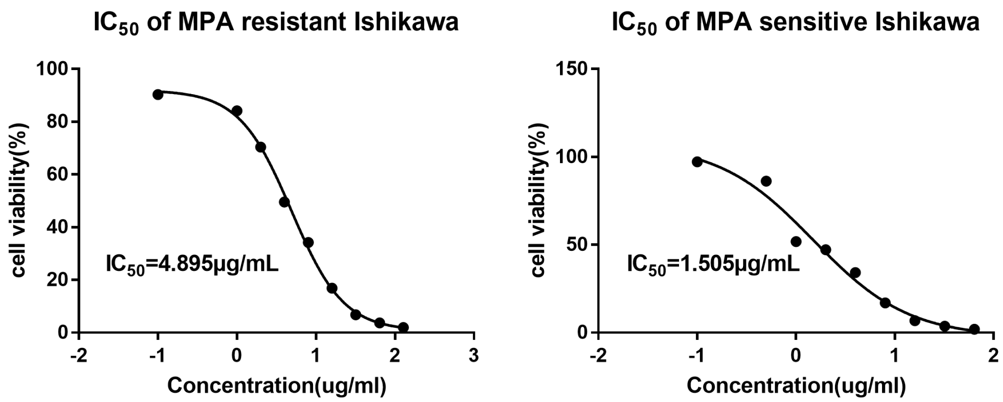

Figure S4. The protein expression of FBW7 in Ishikawa-MPA/shFBXL16 and Ishikawa-MPA/shNC cells by immunoprecipitation detection.

Table S1 Primers for genes

Gene	Sequence (5'->3')	Template strand
**FBXL16**	Forward primer	AGCGGCCTTATTTATCTGGGC
	Reverse primer	GACATCTTCCTGGCACGCTC
**cyclin D1**	Forward primer	GATGCCAACCTCCTCAACGA
	Reverse primer	GGAAGCGGTCCAGGTAGTTC
**cyclin E1**	Forward primer	CCATCATGCCGAGGGAGC
	Reverse primer	TAATCCGAGGCTTGCACGTT
**GAPDH**	Forward primer	GACAGTCAGCCGCATCTTCT
	Reverse primer	GCGCCCAATACGACCAAATC
